# Health Behaviors of Chinese Childhood Cancer Survivors: A Comparison Study with Their Siblings

**DOI:** 10.3390/ijerph17176136

**Published:** 2020-08-24

**Authors:** Carmen W. H. Chan, Kai Chow Choi, Wai Tong Chien, Janet W. H. Sit, Rosa Wong, Karis K. F. Cheng, Chi Kong Li, Hui Leung Yuen, Chi Keung Li

**Affiliations:** 1 The Nethersole School of Nursing, The Chinese University of Hong Kong, Hong Kong, China; kchoi@cuhk.edu.hk (K.C.C.); wtchien@cuhk.edu.hk (W.T.C.); janet.sit@cuhk.edu.hk (J.W.H.S.); 2 Department of Paediatrics and Adolescent Medicine, The University of Hong Kong, Hong Kong, China; szemanwg@hotmail.com; 3 Alice Lee Centre for Nursing Studies, National University of Singapore, Singapore 119077, Singapore; nurckfk@nus.edu.sg; 4 Department of Paediatrics, The Chinese University of Hong Kong, Hong Kong, China; ckli@cuhk.edu.hk; 5 Department of Paediatrics, Queen Elizabeth Hospital, Hong Kong, China; yuenhl@ha.org.hk; 6 Department of Paediatrics & Adolescent Medicine, Princess Margaret Hospital, Hong Kong, China; lichik@netvigator.com

**Keywords:** childhood cancer survivor, health behavior, health insurance, life insurance, psychological distress, health-related quality of life

## Abstract

*Purpose:* This study aimed to compare health behaviors between the childhood cancer survivors (CCS) and their sibling controls and to examine the pattern of health behaviors of the Hong Kong Chinese CCS and its associations with their health-related quality of life and psychological distress. *Methods:* A cross-sectional telephone survey was conducted. A total of 614 CCS and 208 sibling controls participated in this study. Patterns of health behaviors including lifestyle behaviors, cancer screening practices, and insurance coverage were compared. Multivariate regression analyses were performed for examining factors associated with health behaviors in CCS. *Results:* CCS had less alcohol consumption when compared with their sibling controls (adjusted odds ratio (AOR) = 0.65, *p* = 0.035). The sibling controls were more likely to have cancer screening practices (AOR = 0.38, *p* = 0.005) and health (AOR = 0.27, *p* < 0.001) and life insurance coverage (AOR = 0.38, *p* < 0.001). Among the CCS, those who were male, having a job or higher education, shorter time since diagnosis, and type of cancer suffered were significantly associated with alcohol consumption. Those CCS who were drinkers indicated poorer mental health (*p* = 0.004) and more psychological distress. Female CCS undertaking cancer screening were more likely to be employed, married/cohabiting, and have received intensive cancer treatment. *Conclusion:* This study reveals that Chinese childhood cancer survivors are less likely to engage in unhealthy lifestyle behaviors, insurance coverage and cancer screening, when compared with their siblings. *Implications for Cancer Survivors*: It is crucial for healthcare professionals to identify strategies or target interventions for raising CCS’s awareness of their cancer risks and healthy lifestyle throughout their life.

## 1. Introduction

Survival after childhood cancer now exceeds 80% throughout the US, Europe, and Australia [1,2,3], and between 40% and 58% in Shanghai, China [1]. Childhood cancer survivors (CCS), similar to other adolescents and young adults, often face wide varieties of short- and/or long-term health and psychosocial issues, are at increased health risk, and face challenges in relation to lifestyle behaviors [4]. For CCS, stress and physical discomfort associated with cancer and its treatment may pose additional challenges and increase their unhealthy behaviors [5]. Health-risk behaviors such as smoking and drinking can further increase survivors’ vulnerability to health problems. It has been reported that CCS are at heightened risk for other chronic health problems such as obesity, diabetes, and cardiovascular diseases [6]. Reducing/modifying unhealthy behaviors plays a key role in controlling the morbidity and mortality rates among adolescents and young adults [7,8].

In recent years, an increasing amount of research has been done to examine the patterns of CCSs’ health behavior in different populations worldwide. Young British adult survivors of childhood cancer were more likely to engage in healthy lives than age- and sex-matched controls or siblings [9]. In Australia and Korea, CSSs were found to have fewer substance use behaviors compared to their healthy peers [10,11]. In the United States, however, survivors were found to have a greater risk of being a current smoker [12]. Hence, the health behavior patterns of CSSs are not universal across populations, and there is a need of research to know more about the long-term health behaviors of CSSs in the Chinese population.

A recent review found that CCSs have a higher risk for cancer recurrence or developing subsequent malignant neoplasms [13] and have a greater than twofold increased solid tumor risk extending beyond the age of 40 years as compared with the general population [14]. With the goal of detecting subsequent malignant neoplasms at an earlier and more treatable stage, early initiation of routine cancer screening and surveillance is needed. However, despite the availability of well-established cancer surveillance guidelines, many CCS have suboptimal adherence with the recommended screening [15]. Their non-compliance behavior may influence the cancer screening and prevention practices of their siblings. When a family has a cancer history, siblings of cancer survivors are also more vulnerable to cancers and require routine screening.

It was found that many CCS were uninsured or had difficulties in obtaining insurance [16]. Lack of health insurance may further impede cancer screening practices in this survivor population [17]. It is therefore important to know whether there are subgroups of survivors who experience more barriers to follow-up care and cancer screening. The information will help to guide policymaking and service planning and ultimately to reduce the future health cost and burden related to childhood cancer.

To our knowledge, this study was the first to examine the health behavior patterns of CSSs and siblings in the Chinese population. The knowledge of risk factors and risk groups of an unhealthy lifestyle can be used to develop a lifestyle intervention to influence modifiable risk factors, while unmodifiable risk factors can be used to identify subgroups at risk. We sought to (1) compare patterns of health behaviors between Hong Kong Chinese CCS and their sibling controls; (2) examine factors associated with those health behaviors of CCS that were found different from their sibling controls; (3) examine the association of CCS’ health behaviors with their HRQoL and psychological distress.

## 2. Methods

### 2.1. Study Design and Sample

A cross-sectional telephone survey was conducted. The electronic medical record system (EMS) of adolescent and young adult survivors of childhood cancer in pediatric departments of three regional hospitals in Hong Kong were accessed with approval granted. Childhood cancer survivors (CCS) who met the inclusion criteria below were invited to participate using convenience sampling. The inclusion criteria for CCS were: (1) having a diagnosis of childhood cancer prior to age 19, according to the guideline of the International Classification of Childhood Cancer [18]; (2) had received childhood cancer treatments at any one of the three regional hospitals in Hong Kong; (3) off-treatment for 2 years or more; (4) survival of at least 5 years from the time of diagnosis; and (5) Chinese speaking and aged 18 or above. Nearest age (>16 years old) siblings of eligible survivors were invited to participate in a comparison group. Sibling controls were used in 17 studies of CCS and were considered as the most relevant group and effective method for comparing of the survivors’ health behaviors or outcomes [19].

There were around 800 eligible CCSs in the EMS of the three regional hospitals. To enhance the representativeness of the study sample, we aimed to recruit at least three-quarters of them. A total of 614 CCSs were finally recruited, of which gender-specific and cancer (diagnosis)-specific subgroups were included according to the Hong Kong Cancer Registry statistics. One hundred and twelve survivors did not have any siblings. For the control subjects, we could only recruit 208 sibling controls at a response rate of 41%. Nevertheless, a sample size of 614 CCSs and 208 sibling controls could achieve 80% of study power to detect an effect size of as small as 0.22 for continuous variables at a two-sided 5% level of significance and odds ratios of 1.58 to 1.98 for binary variables at a one-sided 2.5% level of significance. All procedures performed in this study involving human subjects were approved by the Joint CUHK-NTEC Clinical Research Ethics Committee (CRE-2008.220) and in accordance with the Declaration of Helsinki. Written consent was obtained from participants and/or their parents prior to undertaking the telephone survey.

### 2.2. Study Instrument

#### 2.2.1. Baseline Questionnaire

The research team adopted and modified the Baseline Questionnaire developed by the Childhood Cancer Survivor Study (CCSS) of the University of Minnesota [20]. This questionnaire was translated into Chinese and was used to collect information on demographic, health history, and health behavior. An expert panel that involved researchers, oncologists, oncology nurses, CCSs, and their siblings reviewed the Chinese questionnaire and established its content validity. The questionnaire was used in previous studies [20,21] involving adolescent and adult survivors of childhood cancer.

#### 2.2.2. The Chinese Version of the SF-36

The SF-36, frequently used instrument for assessing health-related quality of life (HRQoL) in childhood cancer survivors, was adopted [19]. It includes 36 items designed to measure eight dimensions of HRQoL, namely physical functioning, general mental health, role limitation due to a physical health problem, bodily pain, social functioning, role limitations due to emotional problems, vitality, and general health perceptions. The SF-36 has established psychometric properties when used in long-term CCSs (age 16 or above) [22]. The Chinese version was validated by Lam et al. [23], and the normative values of the SF-36 for the Hong Kong general population and different age/sex groups were reported [24].

#### 2.2.3. Brief Symptom Inventory (BSI)

Psychological screening of cancer survivors was conducted using the Chinese version of the BSI-18. This 18-item self-reported symptom checklist was designed to measure three dimensions of psychological distress, including somatization, depression, and anxiety. A cut-off score of >50 is used for identifying potentially distressed persons. The validity testing conducted by Recklitis et al. [25] supports the strong diagnostic utility of BSI-18 for adult survivors of childhood cancer. The BSI-18 was adopted in a study of the Chinese population by Wang et al. [26], and the normative values of the healthy US populations and oncology patients were reported.

### 2.3. Data Collection Methods

CCS samples were drawn from the electronic medical records of the three participating regional hospitals in Hong Kong according to the sampling criteria. These participating hospitals provided treatment and services for 60% of childhood cancer patients in Hong Kong. Invitation letters, explaining the study purpose and nature of participation, were sent to the eligible individuals and their siblings. The survivors helped to ask their siblings for their willingness to participate and then gave the research team the siblings’ contact address. Not all survivors had siblings and some siblings refused to participate. We only approached those siblings who agreed to participate. A consent form with returned envelop was enclosed in the invitation letter. After receiving the signed written consent form, the interviewers called the participants and conducted telephone interviews using the structured questionnaires, including SF-36, BSI-18, and socio-demographic and clinical data. During the interviews, CCSs were asked to answer the whole set of questionnaires while their siblings were asked to answer SF-36, BSI-18, and socio-demographic data. Telephone interviews were justified because of a shorter completion time and better response rate. For ‘no answer’ cases, to facilitate participation, the interviewers did at least three phone calls on different days of the week and different hours of the day from the original call. If the phone number retrieved from hospital record could not reach the participant, work/mobile numbers or parents’ home numbers were called. To ensure the validity of the interviews, a research nurse made phone calls to a randomly selected 5% of the participants to double check the answers in the questionnaires were truly provided by the participants.

### 2.4. Data Analysis

Data analyses were performed using the IBM SPSS 24.0 (IBM Crop., Armonk, NY). Appropriate descriptive statistics such as frequency, percentage, and mean (SD) were used to calculate and summarize socio-demographic and disease characteristics, as well as outcome variables. Independent t-tests or chi-square (or Fisher’s exact) tests, where appropriate, were performed to examine differences on socio-demographic and disease characteristics between CCSs and sibling controls.

Multivariate logistic regressions were performed to compare health behavior outcomes between CCSs and sibling controls with adjustment for socio-demographic characteristics. Follow-up analyses were conducted to identify socio-demographic and disease characteristics among CCSs associated with the differences in health behaviors that were found different from their sibling controls using multivariate logistics regression. Further sub-group analyses were conducted to examine whether the CCSs’ HRQoL and psychological distress differed in terms of their patterns of health behaviors, adjusting for their socio-demographic and disease characteristics. Potential confounding variables in these models included age, age at diagnosis of cancer, gender, type and severity of cancer, and type and length of cancer treatment. All statistical tests were two-sided with the level of significance set at 0.05.

## 3. Results

The socio-demographic and disease characteristics of the 614 CCSs and 208 siblings are shown in Table 1. Survivors (mean age: 24.0 ± 5.1 years) were significantly older than the siblings (mean age: 21.9 ± 5.6 years). Compared with the sibling comparison group, a significantly greater proportion of survivors were male, single, less educated, currently unemployed, living on a lower personal monthly income, and not a housing owner. The majority of survivors were diagnosed of leukemia (45%), followed by bone and soft tissue cancer (12%) and lymphoma (10%).

### 3.1. Health Behaviors

The comparisons between the survivors and sibling groups on health behaviors are shown in Table 2. There was no difference in smoking rates between the CCSs and siblings. Drinking rates were lower in the CCSs than their siblings (adjusted odd ratio, AOR = 0.65, *p* = 0.035). On the other hand, the siblings were more likely to have health (AOR = 0.27, *p* < 0.001) and/or life insurance coverage (AOR = 0.38, *p* < 0.001) than the CCSs. In particular, compared with female siblings, a significantly smaller proportion of female CCSs had ever taken a pap smear test (AOR = 0.38, *p* = 0.005) and breast examination (AOR = 0.54, *p* = 0.033).

Follow-up analyses using multivariate logistic regression were conducted to identify the CCSs’ socio-demographic and disease characteristics associated with the differences in health behaviors that were found different from their sibling controls (Table 3 and Table 4). Results showed that the odds of having alcohol consumption, health insurance coverage and life insurance coverage were all increased generally with educational level. The odds of having health insurance coverage were also significantly increased with years since cancer diagnosis (AOR = 1.06, *p* = 0.028). Those CCSs not born in Hong Kong were less likely to have life insurance coverage (AOR = 0.39, *p* = 0.044). Other demographic and disease risk factors significantly associated with alcohol consumption among survivors included gender (female CCS was less likely to have alcohol consumption compared with male CCS, AOR = 0.65, *p* = 0.038), being employed as compared with those unemployed (AOR = 4.47, *p* < 0.001), as well as type of cancer (of note, those who had brain and central nervous system (CNS) malignances were less likely to have alcohol consumption compared with those who had leukemia. For female survivors, no socio-demographic characteristics were found significantly associated with pap smear test and breast examination although those married/cohabiting and having job/better income/housing ownership were more likely to have had cancer screening tests. However, the uptake of these screening tests might be influenced by their previous cancer treatments received. In general, those treated with more intensive methods including chemotherapy and radiotherapy, as well as chemotherapy, surgery, and radiotherapy, were more likely to have ever undergone a pap smear test and breast examination.

### 3.2. Health-Related Quality of Life and Psychological Distress by Health Behavior Status

Further post hoc analyses were conducted to examine whether the CCSs’ HRQoL and psychological distress levels differed by their patterns of health behaviors. Results of linear regression analyses with adjustment for the socio-demographic and cancer history characteristics listed in Table 1 revealed that the CCSs who were drinkers had poorer mental health (*p* = 0.004) and more psychological distress, particularly in the dimensions of depression (*p* = 0.038) and somatization (*p* = 0.045), than non-drinkers (Table 5). Health and life insurance coverage did not appear to impact on the CCSs’ HRQoL and psychological distress (Table 6 and Table 7). Female survivors who had ever undergone pap smear and/or breast examination reported a significantly higher level of somatization (*p* = 0.001 and *p* = 0.025, respectively) than those who had never taken the tests (Table 8 and Table 9).

## 4. Discussion

Smoking, alcohol consumption, and a low level of routine cancer screening are key health behaviors related to increased risks of developing chronic health problems, including subsequent malignant neoplasms [14]. Studies evaluating health behavioral practices among CCSs are important because many of these poor health behaviors are modifiable and have the potential to be ameliorated through interventions [4,14]. Results from this population-based study found that the CCSs in Hong Kong, when compared with their sibling controls, had less alcohol consumption, less cancer screening practices (including pap smear and/or breast examination), and low rates of health and life insurance coverage.

### 4.1. Health Behaviors

The finding of lower alcohol consumption in CCS is consistent with previous studies that CCSs were less likely to be current and heavy drinkers, when compared with sibling controls (43.9% versus 66.8%) [27,28]. Several sociodemographic and disease-related factors appear to influence CCSs’ rates of alcohol consumption in this study. The findings suggest that male survivors who are employed and also those who are more educated were associated with an increased odds of alcohol consumption. This is somewhat different from the findings of the cross-sectional Childhood Cancer Survivor Study, which reported that attaining secondary education or below was a risk factor for heavy drinking [27]. Our findings suggest that as educated CCSs generally work in higher wage job sectors such as business firms requiring occasional drinking, this job requirement may make it difficult for CCSs to be totally abstinent from alcohol. Another possibility is that these CCSs were part of a higher social class in which social drinking is prevalent. Hence, it is important to educate this CCS population about the importance of controlling their drinking within the level recommended for good health. 

By contrast, we observed a low proportion (less than 10%) of current smokers in this study; and no significant differences in smoking rates were found between the CCSs and sibling controls. These findings are in line with previous studies [4,29]. Significant difference in alcohol use but not cigarette use between CCSs and siblings is interesting because a vast body of literature documented that smoking usually co-occurs with drinking [30]. The question of why some survivors would engage in alcohol use more frequently than cigarette use deserves further investigation. Social drinking might be considered less risky to health as compared to smoking by CCSs. The public education and awareness program targeted more the harmful effect of smoking, while the risk of drinking was not emphasized. Only recently, the WHO stated alcohol is carcinogenic. It is worth noting that large scale cohort studies in the US identified a 1.13-fold increased risk of alcohol-related cancer in women who had never smoked, with mild to moderate alcohol drinking [31]. Cancer risks associated with the consumption of alcoholic beverages need health professional’s further attention.

More importantly, the CCSs who were ever drinkers reported poorer mental health and higher psychological distress, particularly in somatic symptoms and depression, when compared with non-drinkers. The associations between poor mental health and problem drinking engagement was documented in previous studies [4,32]. Previous CCS studies identified that CCSs with depression (AOR = 1.7, *p* < 0.001), somatization (AOR = 1.7, *p* < 0.001), and/or cancer-related anxiety (AOR = 1.2, *p* < 0.05) were at an increased risk of binge or heavy drinking [27]. Experience of substantial stressful life events and feeling more susceptible to the late effects of cancer were also seen to be associated with alcohol use in CCSs [33,34]. Elevated stressors due to cancer and treatment may further exacerbate CCSs’ dependence on alcohol as a stress-relieving practice. Regular evaluation of these survivors’ psychological state would allow for timely support and interventions before the onset of problematic alcohol drinking.

### 4.2. Cancer Screening

The majority of the male participants (87.9% among CCSs and 89.5% among their sibling controls) had rarely or never performed testicular self-examination. No significant difference was found between the male CCSs and sibling controls. Our findings are comparable with previous studies [35,36]. This is not unexpected as testicular cancer is rare as second cancer in CCSs, thus clinicians rarely mention this health practice to CCSs, and there is also no public awareness program in Hong Kong. Similarly, the majority of all female respondents had not undertaken pap smear test and breast examination. In a subgroup analysis, we found a statistically significant higher pap smear update rate in the sibling controls (32.1%) than in female CCSs (10.4%). This may be due to the recommendation given by the Government of the Hong Kong Special Administrative Region that women who are aged 25 or above with sexual experiences should begin regular cervical smears [4]. Given that most of our CCSs were young adults (average age for CCSs: 21.6 years and siblings: 24.0 years), many CCS respondents had not reached the recommended age for taking their first pap smear test at the time of our survey. The fact that the siblings were generally older than the CCSs could also be a possible reason for the higher pap smear uptake rates in the siblings. Female siblings might be more health conscious when there was a family member with a history of cancer, thus, leading to higher screening rate for pap smear. Other possible reasons are that CCS might have underestimated their risk of developing another cancer and/or the importance of routine cancer screening.

A statistically significant higher proportion of the sibling controls reported having taken a breast examination than female CCSs (39.3% and 25% respectively), suggesting the siblings might have greater breast cancer awareness than the CCSs. Follow-up analysis did not identify any socio-demographic factors significantly influencing female CCSs’ compliance with cancer screening for both pap smear and breast examination. Nevertheless, female CCSs being married or cohabiting, currently engaging in paid work, who had higher income/housing ownership, and had undertaken intensive combination of cancer treatments (chemotherapy, surgery, and radiotherapy) tend to be more compliant with cancer screening. Female CCSs with sexual activities might be more aware of the risk of cervical cancer and thus opted for screening. CCSs with a history of intensive anti-cancer treatment might be more health conscious and willing to participate in screening. The results indicate a need for raising cancer awareness and its prevention practices among female CCSs, especially for those having a higher chance of non-compliance with screening. 

This study also found greater distress regarding somatic symptoms in CCSs who had ever done pap smear and/or breast examination possibly because of their heightened awareness of unusual health conditions. It is possible that the screened survivors could be prompted by the signs and symptoms related to a disease to attend screening; thus, these survivors reported greater psychological distress. On the other hand, unscreened survivors might have considered attending screening to be associated with sickness rather than preventive health [37]. This misconception would reduce CCSs’ motivation to perform routine screening [38]. Misconceptions about childhood cancer are common. Because of the rarity of childhood cancer, some health care providers are not knowledgeable about the cancer-related risks and risk-reduction methods relevant to this population [39], which may further reduce information sharing among the CCSs, providers and family members. Therefore, there is also a need for enhancing health care providers’ knowledge of childhood cancer and in turn improve the quality of care for CSSs. 

### 4.3. Insurance Coverage

In our study, over 70% of the CCSs did not have health or life insurance coverage. Lack of insurance coverage in the CCSs was associated with low educational attainment and more recent cancer diagnosis. Many CCSs cannot have health insurance coverage because of their medical history [40]. Previous studies also reported lower rates of health insurance in CCSs in comparison with their sibling controls [41,42,43]. Consistently, our study found that the CCSs were less likely to be covered by health and life insurance than their siblings. From a research brief published by the Hong Kong government in 2018, 42% of Hong Kong citizens are protected by health insurance [44]. The percentage for the elderly, aged 65 or above, is only 10.4%. The percentage of insurance coverage by siblings appears to be comparable to the general public, while that of survivors is lower. The result is alarming because uninsured CCSs would likely have difficulties in obtaining access to health care services [45] such as cancer screening and preventive treatment and thus might feel more worried about future health [40]. Even with insurance coverage, it is usually offered at a high premium, which can be unaffordable for most CCSs, particularly those who are socio-economically disadvantaged because of their age and unemployment. Nevertheless, Hong Kong adopts a national health system where pap smears and mammograms are provided by the government at an affordable cost. The HPV vaccine is rather expensive and will not be covered by any insurance plan. Thus, education of CCSs on potential second cancer and advise on the screening program is more important than purchasing insurance in the Hong Kong context.

### 4.4. Limitations

Despite the use of population-based data, this study has several limitations. Firstly, different behaviors were assessed with only one question for each behavior, such as alcohol consumption (yes/no) and smoking (yes/no), we did not ask the participants about the amount and frequency of alcohol use. Therefore, we could not ascertain of the rates of problematic drinking in both the CCSs and their siblings. We did not collect information on family medical history, which could influence the health behavior patterns of the CCS and siblings. Our cross-sectional analyses could not distinguish cause and consequences. Quality of life and psychological distress can be interpreted both as risk factor and consequence of an unhealthy lifestyle behavior. Moreover, the sample size of siblings was lower, and their data may not be representative. Lastly, participants’ self-reports of health-related quality of life and psychological distress could involve a response bias.

## 5. Conclusions

This study reveals that Chinese childhood cancer survivors are less or equally likely to engage in unhealthy lifestyle behaviors, insurance coverage, and cancer screening, when compared with their (healthy) siblings. Socioeconomically disadvantaged survivors are particularly at risk for not taking adequate preventive measures. Our findings also provide support for the need of interventions targeting CCSs for enhancing awareness of their health risks and health behaviors to ameliorate modifiable risks. More studies should be conducted to identify facilitators and barriers to healthy lifestyle and its maintenance among CCSs and establish a long-term follow-up program.

## Figures and Tables

**Table 1:** Socio-demographic and cancer history characteristics of the participants.

Characteristics	Siblings (*n* = 208)	Survivors (*n* = 614)	*p*-Value
Socio-demographics			
Age (years) ^†^	24.0 (5.1)	21.9 (5.6)	<0.001
Gender			
Male	94 (45.2%)	360 (58.6%)	0.001
Female	114 (54.8%)	254 (41.4%)	
Birth in Hong Kong			
Yes	189 (91.3%)	533 (87.1%)	0.105
No	18 (8.7%)	79 (12.9%)	
Marital status			
Single	177 (86.3%)	571 (93.1%)	0.001
Married/cohabiting	22 (10.7%)	40 (6.5%)	
Divorced/widowed/separated	6 (2.9%)	2 (0.3%)	
Educational level			
Not completed secondary	24 (11.5%)	259 (42.5%)	<0.001
Completed secondary	39 (18.8%)	177 (29.1%)	
Sub-degree	42 (20.2%)	78 (12.8%)	
University or above	103 (49.5%)	95 (15.6%)	
Employment status			
Unemployed	60 (29.0%)	301 (49.2%)	<0.001
Employed	147 (71.0%)	311 (50.8%)	
Personal monthly income (HK$)			
No income	68 (32.9%)	312 (51.1%)	<0.001
1–9999	60 (29.0%)	160 (26.2%)	
10,000–19,999	53 (25.6%)	102 (16.7%)	
≥20,000	26 (12.6%)	36 (5.9%)	
Housing ownership			
No	163 (78.4%)	567 (92.3%)	<0.001
Yes	45 (21.6%)	47 (7.7%)	
Disease characteristics			
Years since diagnosis ^†^		14.1 (6.8)	
Types of cancer			
Leukemia		279 (45.4%)	
Lymphoma		62 (10.1%)	
Bone and soft tissue cancers		75 (12.2%)	
Brain and CNS malignancies		52 (8.5%)	
Others		146 (23.8%)	
Treatments received			
Chemotherapy/Surgery/Radiotherapy only		241 (46.4%)	
Chemotherapy & surgery		145 (27.9%)	
Chemotherapy & radiotherapy		62 (11.9%)	
Chemotherapy & surgery & radiotherapy		71 (13.7%)	

Data marked with ^†^ are presented as mean (standard deviation), all others are frequency (percentage).

**Table 2:** Comparisons between childhood cancer survivors and their siblings on health behavior outcomes.

Health Behavior Outcomes	Unadjusted Comparison				Adjusted Comparison	
	Siblings (ref)	Survivors	OR_U_	*p*-Value	OR_A_ (95% CI)	*p*-Value
Among all respondents						
Alcoholic consumption						
No	69 (33.2%)	343 (56.1%)				
Yes	139 (66.8%)	268 (43.9%)	0.39	<0.001	0.65 (0.43–0.97)	0.035
Smoking						
No	189 (90.9%)	557 (90.7%)				
Yes	19 (9.1%)	57 (9.3%)	1.02	0.949	0.85 (0.44–1.66)	0.638
Health insurance coverage						
No	86 (41.3%)	464 (76.6%)				
Yes	122 (58.7%)	142 (23.4%)	0.22	<0.001	0.27 (0.18–0.39)	<0.001
Life insurance coverage						
No	103 (50.0%)	476 (78.7%)				
Yes	103 (50.0%)	129 (21.3%)	0.27	<0.001	0.38 (0.26–0.56)	<0.001
Among all male respondents						
Monthly testicular self-examination						
Rarely or never	77 (89.5%)	299 (87.9%)				
Occasionally/regularly	9 (10.5%)	41 (12.1%)	1.17	0.682	1.28 (0.54–3.02)	0.573
Among all female respondents						
Monthly breast self-examination						
Rarely or never	66 (59.5%)	174 (69.3%)				
Occasionally/regularly	45 (40.5%)	77 (30.7%)	0.65	0.068	0.87 (0.50–1.51)	0.620
Ever had pap smear test						
No	76 (67.9%)	225 (89.6%)				
Yes	36 (32.1%)	26 (10.4%)	0.24	<0.001	0.38 (0.19–0.75)	0.005
Ever had breast examination						
No	68 (60.7%)	189 (75.0%)				
Yes	44 (39.3%)	63 (25.0%)	0.52	0.006	0.54 (0.31–0.95)	0.033
Mammogram						
No	105 (94.6%)	237 (94.0%)				
Yes	6 (5.4%)	15 (6.0%)	1.11	0.837	2.31 (0.68–7.84)	0.181

Ref: siblings were set as the reference group for estimating the odds ratios of survivors vs. siblings on the outcomes; OR_U_: unadjusted odds ratio of survivors vs. sibling (ref); OR_A_: odds ratio adjusted for age, gender (except for those gender-specific outcomes), birthplace, martial status, educational level, employment status, personal income, and housing ownership obtained by multivariable logistic regression.

**Table 3:** Logistic regression on the associations between socio-demographic and cancer history characteristics and health behavior outcomes (that found a significant difference between the childhood cancer survivors and their siblings) among the childhood cancer survivors (*n* = 614).

Characteristics	Alcoholic Consumption		Health Insurance Coverage		Life Insurance Coverage	
	Odds Ratio (95% CI)	*p*-Value	Odds Ratio (95% CI)	*p*-Value	Odds Ratio (95% CI)	*p*-Value
Socio-demographics						
Age (years)	1.03 (0.97–1.10)	0.373	0.90 (0.84–0.97)	0.006	0.96 (0.90–1.02)	0.203
Gender						
Male (ref)	1		NE	NE	NE	NE
Female	0.65 (0.43–0.98)	0.038				
Birth in Hong Kong						
Yes (ref)	NE	NE	1		1	
No			0.61 (0.28–1.34)	0.221	0.39 (0.16–0.98)	0.044
Marital status						
Single/divorced/widowed/separated (ref)	1		1		1	
Married/cohabiting	0.67 (0.29–1.56)	0.356	1.71 (0.70–4.19)	0.240	0.87 (0.36–2.09)	0.756
Educational level						
Not completed secondary (ref)	1		1		1	
Completed secondary	2.73 (1.54–4.84)	0.001	1.28 (0.63–2.60)	0.491	0.83 (0.40–1.70)	0.604
Sub-degree	3.67 (1.80–7.48)	<0.001	1.43 (0.61–3.38)	0.411	1.09 (0.46–2.56)	0.844
University or above	4.00 (1.92–8.32)	<0.001	3.07 (1.31–7.16)	0.010	1.60 (0.71–3.61)	0.259
Employment status						
Unemployed (ref)	1		1		1	
Employed	4.47 (2.32–8.62)	<0.001	0.91 (0.41–2.03)	0.820	0.91 (0.40–2.05)	0.810
Personal monthly income (HK$)						
No income (ref)	1		1		1	
1–9999	1.03 (0.57–1.88)	0.915	1.64 (0.83–3.21)	0.153	1.62 (0.80–3.30)	0.181
10,000–19,999	1.05 (0.50–2.21)	0.896	1.82 (0.77–4.30)	0.175	3.23 (1.39–7.53)	0.006
≥20,000	4.34 (1.20–15.75)	0.026	1.12 (0.37–3.37)	0.839	3.63 (1.30–10.18)	0.014
Housing ownership						
No (ref)	1		1		1	
Yes	1.51 (0.68–3.37)	0.315	1.54 (0.64–3.67)	0.334	1.44 (0.67–3.09)	0.351
Disease characteristics						
Years since diagnosis	0.95 (0.91–0.99)	0.020	1.06 (1.01–1.12)	0.028	1.04 (0.99–1.10)	0.094
Types of cancer						
Leukemia (ref)	1		1		1	
Lymphoma	1.42 (0.70–2.87)	0.334	2.14 (1.04–4.40)	0.040	0.90 (0.43–1.87)	0.777
Bone and soft tissue cancers	1.52 (0.80–2.89)	0.199	1.44 (0.61–3.40)	0.409	0.50 (0.20–1.28)	0.148
Brain and CNS malignancies	0.28 (0.11–0.70)	0.007	1.69 (0.68–4.22)	0.259	0.78 (0.32–1.90)	0.581
Others	1.46 (0.86–2.46)	0.162	1.48 (0.75–2.90)	0.257	1.59 (0.94–2.71)	0.085
Treatments received						
Chemotherapy/Surgery/Radiotherapy only (ref)	NE	NE	1		NE	NE
Chemotherapy & surgery			0.46 (0.23–0.89)	0.022		
Chemotherapy & radiotherapy			0.40 (0.17–0.90)	0.027		
Chemotherapy & surgery & radiotherapy			1.05 (0.52–2.14)	0.896		

NE: *p* ≥ 0.2 in univariate analysis and not entered into multivariable logistic regression analysis; ref: reference group of the independent categorical variable.

**Table 4:** Logistic regression on the associations between socio-demographic and cancer history characteristics and health behavior outcomes (that found a significant difference between the childhood cancer survivors and their siblings) among the female childhood cancer survivors (*n* = 254).

Characteristics	Ever Had Pap Smear		Ever Had Breast Examination	
	Odds Ratio (95% CI)	*p*-Value	Odds Ratio (95% CI)	*p*-Value
Socio-demographics				
Age (years)	1.04 (0.85–1.26)	0.728	1.01 (0.91–1.12)	0.930
Birth in Hong Kong				
Yes (ref)	NE	NE	NE	NE
No				
Marital status				
Single/divorced/widowed/separated (ref)	1		1	
Married/cohabiting	3.88 (0.85–17.73)	0.081	2.48 (0.72–8.51)	0.150
Educational level				
Not completed secondary (ref)	1		NE	NE
Completed secondary	2.86 (0.22–36.56)	0.419		
Sub-degree	2.69 (0.18–41.16)	0.476		
University or above	1.04 (0.05–21.03)	0.981		
Employment status				
Unemployed (ref)	1		1	
Employed	7.13 (0.53–95.43)	0.138	2.23 (0.80–6.27)	0.127
Personal monthly income (HK$)				
<10,000 (ref)	1		NE	NE
≥10,000	2.29 (0.67–7.81)	0.184		
Housing ownership				
No (ref)	1		1	
Yes	3.76 (0.33–42.42)	0.284	2.82 (0.55–14.41)	0.214
Disease characteristics				
Years since diagnosis	0.97 (0.86–1.09)	0.583	NE	NE
Types of cancer				
Leukemia (ref)	NE	NE	1	
Lymphoma			3.22 (0.85–12.27)	0.087
Bone and soft tissue cancers			3.61 (0.96–13.54)	0.057
Brain and CNS malignancies			1.99 (0.37–10.62)	0.421
Others			3.24 (1.09–9.65)	0.035
Treatments received				
Chemotherapy/Surgery/Radiotherapy only (ref)	1		1	
Chemotherapy & surgery	1.89 (0.40–8.93)	0.421	0.34 (0.12–1.01)	0.052
Chemotherapy & radiotherapy	7.79 (1.25–48.43)	0.028	1.54 (0.40–5.90)	0.528
Chemotherapy & surgery & radiotherapy	5.40 (1.00–29.03)	0.049	4.29 (1.02–17.96)	0.046

NE: *p* ≥ 0.2 in univariate analysis and not entered into multivariable logistic regression analysis; ref: reference group of the independent categorical variable.

**Table 5:** Health-related quality of life and psychological distress outcome measures among childhood cancer survivors by status of alcoholic consumption.

Outcomes	Alcoholic Consumption			Adjusted Comparison		
	No (ref)	Yes	*p*-Value	B	SE	*p*-Value
Health-related quality of life (SF-36)						
Physical functioning subscale score	92.1 (15.4)	94.2 (10.4)	0.047	1.500	1.373	0.275
Role physical subscale score	86.7 (20.4)	88.0 (18.1)	0.418	1.522	2.078	0.464
Body pain subscale score	86.3 (21.2)	83.6 (20.1)	0.111	−3.831	2.309	0.098
General health subscale score	62.6 (19.4)	60.8 (19.8)	0.256	−3.334	2.042	0.103
Vitality subscale score	65.0 (17.9)	60.4 (17.1)	0.001	−3.474	1.893	0.067
Social functioning subscale score	90.1 (17.7)	88.2 (14.9)	0.157	−4.655	1.920	0.016
Role emotional subscale score	89.8 (18.5)	87.1 (18.1)	0.068	−0.464	2.212	0.834
Mental health subscale score	74.1 (15.1)	70.7 (15.7)	0.007	−5.079	1.728	0.003
Physical health summary component score	51.0 (9.2)	51.6 (7.6)	0.428	0.072	0.870	0.934
Mental health summary component score	52.6 (8.0)	50.2 (8.2)	<0.001	−2.721	0.937	0.004
Psychological distress (BSI-18)						
Somatization	42.4 (6.2)	44.1 (7.5)	0.002	1.588	0.788	0.045
Depression	45.3 (8.0)	47.7 (8.4)	<0.001	1.894	0.909	0.038
Anxiety	42.5 (7.9)	44.2 (8.5)	0.013	1.581	0.936	0.092
Global severity index	40.3 (9.5)	43.3 (10.5)	<0.001	2.998	1.138	0.009

B: regression coefficient of status of alcoholic consumption (never drinker as reference) estimated by multivariable regression with adjustment for socio-demographic and cancer history characteristics, including age, gender, birth place, marital status, educational level, employment status, personal monthly income, housing ownership, years since diagnosis of cancer, type of cancer, and treatments received; SE: standard error of B.

**Table 6:** Health-related quality of life and psychological distress outcome measures among childhood cancer survivors by status of health insurance coverage.

Outcomes	Health Insurance Coverage			Adjusted Comparison		
	No (ref)	Yes	*p*-Value	B	SE	*p*-Value
Health-related quality of life (SF-36)						
Physical functioning subscale score	92.7 (14.0)	93.8 (11.7)	0.406	−1.084	1.325	0.414
Role physical subscale score	86.3 (20.6)	90.5 (14.2)	0.006	3.962	1.997	0.048
Body pain subscale score	84.8 (21.2)	85.6 (19.4)	0.710	1.004	2.228	0.652
General health subscale score	61.6 (19.6)	61.8 (19.5)	0.922	−1.349	1.974	0.495
Vitality subscale score	63.0 (17.5)	63.0 (18.4)	0.996	−1.060	1.818	0.560
Social functioning subscale score	88.7 (17.4)	90.7 (13.9)	0.225	1.066	1.865	0.568
Role emotional subscale score	87.9 (19.0)	90.4 (16.4)	0.137	3.007	2.128	0.158
Mental health subscale score	73.0 (15.2)	71.6 (16.4)	0.365	−1.763	1.680	0.295
Physical health summary component score	51.0 (8.9)	51.8 (7.7)	0.330	−0.182	0.840	0.829
Mental health summary component score	51.5 (8.2)	51.6 (8.1)	0.868	0.182	0.912	0.842
Psychological distress (BSI-18)						
Somatization	42.8 (6.9)	44.4 (6.7)	0.019	1.611	0.759	0.034
Depression	46.4 (8.1)	46.1 (8.6)	0.725	0.205	0.875	0.815
Anxiety	43.3 (8.3)	43.0 (8.1)	0.702	0.444	0.903	0.624
Global severity index	41.6 (10.0)	41.8 (10.2)	0.875	0.688	1.102	0.533

B: regression coefficient of status of health insurance coverage (no coverage as reference) estimated by multivariable regression with adjustment for socio-demographic and cancer history characteristics, including age, gender, birth place, marital status, educational level, employment status, personal monthly income, housing ownership, years since diagnosis of cancer, type of cancer, and treatments received; SE: standard error of B.

**Table 7:** Health-related quality of life and psychological distress outcome measures among childhood cancer survivors by status of life insurance coverage.

Outcomes	Life Insurance Coverage			Adjusted Comparison		
	No (ref)	Yes	*p*-Value	B	SE	*p*-Value
Health-related quality of life (SF-36)						
Physical functioning subscale score	92.3 (14.2)	95.5 (9.8)	0.004	2.595	1.413	0.067
Role physical subscale score	86.4 (20.0)	90.7 (16.3)	0.013	5.732	2.129	0.007
Body pain subscale score	84.8 (21.2)	85.6 (19.0)	0.717	2.196	2.382	0.357
General health subscale score	61.4 (19.4)	62.5 (20.2)	0.581	0.374	2.113	0.859
Vitality subscale score	63.4 (17.5)	61.5 (18.3)	0.296	−2.185	1.943	0.261
Social functioning subscale score	88.2 (17.4)	92.7 (12.7)	0.002	5.170	1.981	0.009
Role emotional subscale score	87.5 (19.1)	92.1 (15.3)	0.006	6.146	2.263	0.007
Mental health subscale score	72.6 (15.7)	72.9 (14.9)	0.845	−0.115	1.799	0.949
Physical health summary component score	50.9 (8.8)	52.3 (7.5)	0.088	1.267	0.897	0.158
Mental health summary component score	51.4 (8.3)	52.0 (7.6)	0.439	0.889	0.975	0.362
Psychological distress (BSI-18)						
Somatization	42.9 (6.9)	44.2 (6.7)	0.048	1.415	0.806	0.080
Depression	46.6 (8.4)	45.6 (7.7)	0.245	−0.567	0.927	0.541
Anxiety	43.3 (8.3)	43.1 (8.1)	0.760	0.573	0.957	0.550
Global severity index	41.6 (10.2)	41.8 (9.6)	0.862	0.836	1.169	0.475

B: regression coefficient of status of life insurance coverage (no coverage as reference) estimated by multivariable regression with adjustment for socio-demographic and cancer history characteristics, including age, gender, birth place, marital status, educational level, employment status, personal monthly income, housing ownership, years since diagnosis of cancer, type of cancer, and treatments received; SE: standard error of B.

**Table 8:** Health-related quality of life and psychological distress outcome measures among female childhood cancer survivors by status of Pap test.

Outcomes	Pap Test			Adjusted Comparison		
	No (ref)	Yes	*p*-Value	B	SE	*p*-Value
Health-related quality of life (SF-36)						
Physical functioning subscale score	92.6 (13.4)	92.5 (12.1)	0.968	4.497	3.519	0.203
Role physical subscale score	84.8 (20.9)	83.7 (24.1)	0.791	2.515	5.198	0.629
Body pain subscale score	81.4 (22.3)	84.0 (19.7)	0.573	6.128	6.178	0.323
General health subscale score	60.0 (19.9)	58.2 (19.1)	0.653	2.818	5.172	0.587
Vitality subscale score	62.0 (17.4)	57.2 (15.8)	0.179	0.463	4.524	0.919
Social functioning subscale score	86.9 (18.5)	86.1 (17.4)	0.817	3.317	5.298	0.532
Role emotional subscale score	85.2 (20.2)	91.3 (17.6)	0.136	10.153	5.642	0.074
Mental health subscale score	71.0 (15.3)	73.7 (15.1)	0.403	5.341	4.335	0.220
Physical health summary component score	50.4 (8.7)	49.8 (9.6)	0.754	2.223	2.292	0.334
Mental health summary component score	50.4 (8.3)	50.9 (8.5)	0.769	2.118	2.457	0.390
Psychological distress (BSI-18)						
Somatization	44.3 (6.6)	40.4 (13.8)	0.170	−7.649	2.238	0.001
Depression	46.2 (7.2)	46.7 (8.1)	0.779	0.269	2.059	0.896
Anxiety	44.5 (8.4)	42.2 (7.4)	0.177	−1.754	2.397	0.465
Global severity index	42.9 (9.4)	38.7 (14.9)	0.165	−7.496	2.900	0.011

B: regression coefficient of Pap test (had not ever had Pap test as reference) estimated by multivariable regression with adjustment for socio-demographic and cancer history characteristics, including age, birth place, marital status, educational level, employment status, personal monthly income, housing ownership, years since diagnosis of cancer, type of cancer, and treatments received; SE: standard error of B.

**Table 9:** Health-related quality of life and psychological distress outcome measures among female childhood cancer survivors by status of breast examination.

Outcomes	Breast Examination			Adjusted Comparison		
	No (ref)	Yes	*p*-Value	B	SE	*p*-Value
Health-related quality of life (SF-36)						
Physical functioning subscale score	92.7 (12.1)	92.3 (16.3)	0.820	1.924	2.570	0.455
Role physical subscale score	84.9 (20.3)	84.2 (23.7)	0.834	3.300	3.766	0.382
Body pain subscale score	82.6 (21.5)	78.8 (23.4)	0.232	1.006	4.498	0.823
General health subscale score	60.1 (19.6)	59.3 (20.3)	0.796	1.281	3.752	0.733
Vitality subscale score	60.9 (18.2)	63.8 (14.1)	0.247	3.262	3.303	0.325
Social functioning subscale score	86.6 (18.6)	87.9 (17.8)	0.620	4.731	3.832	0.219
Role emotional subscale score	84.7 (20.2)	89.3 (19.2)	0.116	8.097	4.086	0.049
Mental health subscale score	70.6 (15.8)	73.4 (13.1)	0.207	5.420	3.133	0.085
Physical health summary component score	50.7 (8.3)	49.3 (10.2)	0.273	0.375	1.672	0.823
Mental health summary component score	49.9 (8.7)	52.1 (6.9)	0.075	3.275	1.770	0.066
Psychological distress (BSI-18)						
Somatization	44.6 (6.6)	42.1 (10.2)	0.028	−3.764	1.666	0.025
Depression	46.0 (7.1)	47.0 (7.7)	0.364	0.582	1.492	0.697
Anxiety	44.7 (8.5)	42.8 (7.9)	0.118	−2.008	1.736	0.249
Global severity index	43.1 (9.4)	40.7 (11.9)	0.100	−4.018	2.129	0.061

B: Regression coefficient of breast examination (had not ever had breast examination as reference) estimated by multivariable regression with adjustment for socio-demographic and cancer history characteristics, including age, birth place, marital status, educational level, employment status, personal monthly income, housing ownership, years since diagnosis of cancer, type of cancer and treatments received; SE: Standard error of B.
